# Machine learning and artificial intelligence in liquid biopsy-based early detection of pancreatic cancer: a scoping review

**DOI:** 10.1038/s44276-026-00232-y

**Published:** 2026-05-21

**Authors:** Joy Ku, Meenakshi Singhal, Margaret Burnette, Samar A. Hegazy

**Affiliations:** 1 Carle Illinois College of Medicine, University of Illinois Urbana-Champaign, Urbana, IL USA; 2 University of Illinois Urbana-Champaign, Urbana, IL USA

## Abstract

Pancreatic ductal adenocarcinoma (PDAC) presents as a cancer with an especially poor prognosis, largely due to the challenges surrounding its early diagnosis. Liquid biopsy has emerged as a promising, noninvasive method for screening across a variety of cancers. This approach is limited, however, by the extensive heterogeneity of biological samples, a challenge that teams are looking to address using artificial intelligence (AI) and machine learning (ML) strategies. By harnessing the ability of ML algorithms to extract the most salient features from complex datasets, researchers can identify biomarkers with high predictive value for PDAC. This review explores the current landscape of AI-powered liquid biopsy for early PDAC diagnosis, focusing on specific techniques and their respective degrees of success. Following PRISMA-ScR guidelines, 85 studies were extracted from PubMed and Scopus with a final 18 studies included. The majority of papers utilized blood (*n* = 15) as the source of liquid biopsy, with the remainder analyzing urine, bile, or cyst fluid. Random forests (*n* = 9) and support vector machines (*n* = 7) were the most frequently implemented ML models, while two papers focused on deep learning methods. Limitations include the lack of standardized reporting for model performance metrics and small cohort sizes with non-granular labels.

## Introduction

Pancreatic cancer, typically referring to pancreatic ductal adenocarcinoma (PDAC), is among the most lethal common cancers due to delayed diagnosis and subsequent poor prognosis and survival rates. One of the key contributing factors to this is the nonspecific nature of the disease’s presenting symptoms, which include fatigue, back or abdominal pain, weight loss, and jaundice [1, 2]. Challenges in screening for PDAC are further exacerbated by early metastatic dissemination of PDAC through possible cellular reprogramming mechanisms, as recently highlighted by Opsahl et al. and their investigations in organ-specific premetastatic niche formation [3]. Unfortunately, the incidence of PDAC continues to rise in the United States across all ethnicities in both men and women, with minority women experiencing the most disproportionate increases [4, 5]. PDAC is also projected to become the second-leading cause of cancer-related mortality by 2030 [6]. A large proportion of cases are metastatic or locally advanced at diagnosis, precluding surgical intervention for these patients. Only 15–20% of patients are eligible for resection at presentation, and even with optimal therapy, the 5-year survival rate remains approximately 10% in the United States [7]. Coupled with limited effective treatment options, there is also a lack of available screening strategies. As such, teams across the world are actively investigating potential biomarkers for early detection of PDAC, leveraging artificial intelligence (AI) or machine learning (ML) tools to streamline the process of identifying those with high diagnostic and prognostic value. This scoping review focuses on the most recent developments in this area, first highlighting the main PDAC biomarkers and sources of liquid biopsies being investigated. Then, specific current AI techniques and associated performance metrics are discussed, including current limitations and future potential.

## Background information

### Pancreatic cancer

PDAC patients are more likely to present with advanced disease at initial diagnosis, with roughly 28% exhibiting regional spread to the lymph nodes and 51% with distant metastasis as reported by the National Cancer Institute’s Surveillance, Epidemiology, and End Results Program [8]. The lack of timely detection can be partially attributed to the pancreas’s retroperitoneal location, which leads to a clinically silent malignancy until the neoplasm grows to a significant size that obstructs surrounding ducts. PDAC is also considered genetically aggressive due to its high frequency of driver mutations like KRAS (>90%), and inactivation of key tumor suppressor genes such as CDKN2A, TP53, and SMAD4 [9, 10]. These mutations result in the progression of pancreatic precursor lesions to malignancy associated with rapid local invasion and distant metastatic spread.

With rates of PDAC incidence on the rise, there is a growing need for strategic screening to identify possible cases at earlier timepoints. Current guidelines recommend that individuals who may have a strong family history of pancreatic cancer or genetic syndromes that increase their risk of developing PDAC, along with patients who have pancreatic cysts (intraductal papillary mucinous neoplasms or mucinous cystic neoplasms) be screened [11, 12]. While pancreatic cysts do not necessarily indicate subsequent malignant transformation, the ability to define an at-risk population who should undergo further testing is the first step to shifting the timeline in the detection of PDAC. Current screening measures include performing an endoscopic ultrasound (EUS) or magnetic resonance cholangiopancreatography (MRCP) [13]. However, these can be costly, semi-invasive, and increase the risk of potential harm to patients [14].

### Liquid biopsy

**Fig. 1:**
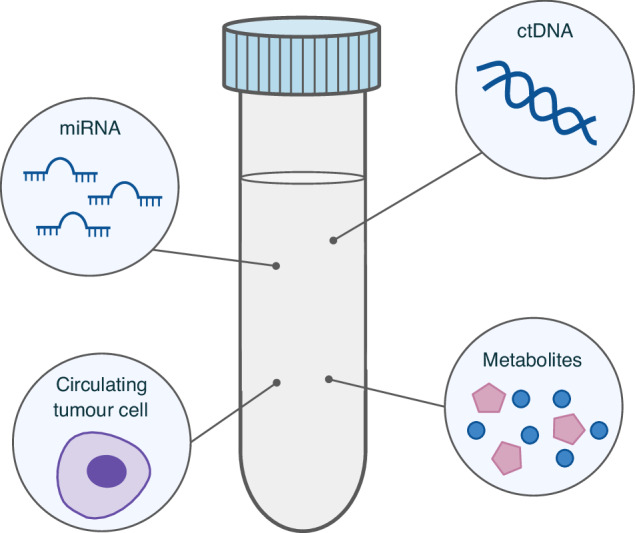
Representation of potential liquid biomarkers found in PDAC patient-sampled biological fluids. This includes ctDNA, miRNA, CTCs, and other cellular metabolites that could be contained in EVs.

Liquid biopsy, the use of readily available biological fluid samples, is an increasingly employed diagnostic approach for cancer biomarker analysis. Liquid biopsies are either non- or minimally invasive and can potentially lower equipment expenses to allow for more routine and accessible screenings. Utilizing liquid biopsy-based testing for PDAC surveillance can mitigate some of the challenges faced by imaging-based screening methods. Carbohydrate antigen 19-9 (CA19-9) is the only currently FDA-approved blood biomarker for PDAC [15]. Diagnostic efficacy of CA19-9 alone remains suboptimal, with its pooled sensitivity, specificity, and AUC at 72%, 86%, and 0.8474, respectively [16]. As such, there is a call for the use of multianalyte panels in conjunction with CA19-9 in order to improve the diagnostic potential of liquid biopsies. Analytes available from liquid biopsies vary widely (Fig. 1), with extracellular vesicles (EVs), microRNAs (miRNAs), circulating tumor DNA (ctDNA), and circulating tumor cells (CTCs), being at the forefront of developments within the field of early cancer detection [17].

EVs are nanoparticles with a lipid bilayer and are secreted by all cell types into surrounding fluids. They display tissue-specific surface protein markers and carry cargo such as proteins, DNA, and RNA (including miRNAs), which allows for analysis of their tissue and cells of origin [18]. Within the context of cancer, tumor-derived EVs are enriched with oncogenic material mirroring the biology of their cell of origin, and their surface markers can allow for localization of the tumor source [19, 20]. Being readily present in blood and other biological fluids, this makes EVs a valuable resource for noninvasive cancer detection.

miRNAs are small, non-coding RNA molecules that can be found in EVs, bound to other proteins, or circulating freely in biological fluids. They regulate gene expression post-transcriptionally, typically either by direct degradation of target mRNA or by reducing translation [21]. Specific miRNAs can be dysregulated in cancer cells, and their stability within biological fluids means that they can serve as promising biomarkers for the detection of cancer-specific expression patterns, especially within PDAC [22].

ctDNA is a subset of circulating cell-free DNA (ccfDNA) that is shed by tumor cells into biological fluids. As small fragments derived directly from tumor cells, they provide insight into any tumor-specific genetic and epigenetic alterations—crucial information for patients with PDAC due to its genetically aggressive etiology [23]. Similarly, circulating tumor cells (CTCs) are intact cancer cells that detach from primary or metastatic tumors and enter the bloodstream. Apart from cancer detection, they also provide direct representation of the tumor and allow for comprehensive analysis of the main tumor from its genetics to proteomics, potentially indicating disease burden and prognosis of PDAC [24, 25]. Both ctDNA and CTCs can be present without metastasis of the primary tumor, with several teams having demonstrated their significance in the detection of early-stage PDAC [26–28].

### AI/ML use for liquid biopsy

**Fig. 2:**
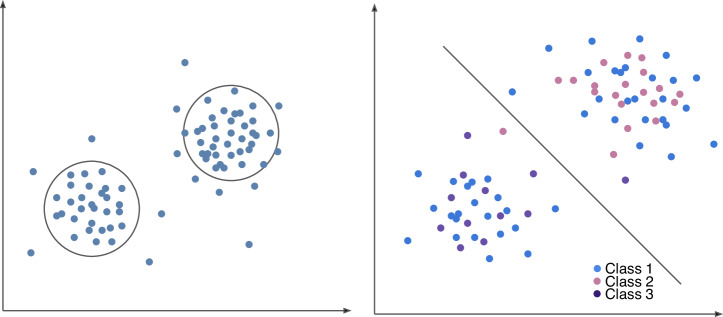
Visual representation of machine learning when applied to different data types (unlabeled versus prelabeled). **A** Unsupervised learning to detect patterns on unlabeled data; **B** Supervised learning on prelabeled data.

Liquid biopsy samples are highly heterogeneous populations (Fig. 1), with the biomarkers discussed in Section 2.2 (EVs, miRNA, ctDNA, CTCs) presenting in varying concentrations in biological fluids. Given the unique properties of each component (size, biochemical composition, etc.), the nature of the data lends itself well to pattern recognition techniques established within the field of artificial intelligence (AI), such that candidate biomarkers can be identified via classification or clustering algorithms (Fig. 2). AI can be understood as two main approaches: supervised or standard machine learning (ML), and unsupervised or deep learning (DL). Supervised ML involves algorithms that first train on a set of labeled data, before mapping input features from the test dataset to a known set of output labels [29]. ML can carry out either regression or classification tasks on continuous or categorical data. Common algorithms include support vector machines (SVMs), logistic regression (LR), linear discriminant analysis (LDA), and decision tree-based methods like random forests (RF). RF models are composed of an ensemble of multiple decision trees that each make a prediction on a separate portion of the dataset. When combined, these trees provide a more stable result than they would alone. While RFs provide advantages like reducing the potential for model overfitting and informing the importance of individual features, they also pose challenges related to training time and memory usage as tree count increases [30]. SVMs, another common algorithm type, serve to classify data by optimizing the hyperplane, which is a line used to stratify points within an *n*-dimensional space [31]. SVMs can classify both linear and nonlinear data, enabling them to perform well on unstructured information. However, like random forests, SVMs require additional computational resources when scaling to large datasets.

Meanwhile, deep learning is a subset of ML that employs artificial neural networks arranged as combinations of multiple hidden layers and nonlinear functions to learn underlying patterns from unlabeled, multidimensional datasets [32]. DL approaches include convolutional neural networks, transformers, and recurrent neural networks. While these methods may offer superior predictive performance on larger, more complex datasets, there is a tradeoff of reduced feature interpretability due to more abstract extraction of data patterns, which should be acknowledged when looking to synthesize physiological and clinical insights. These methods have been utilized across numerous biomedical studies, ranging from imaging-based breast cancer diagnosis, to predicting outcomes in patients with Alzheimer’s disease, to early prediction of sepsis using free-text electronic health record data [33–35].

AI also provides the computing capacity necessary to design multianalyte panels consisting of several candidate features in order to improve speed, reliability, and diagnostic accuracy. A historical model demonstrating the development pathway of multianalyte panels is prenatal Trisomy 21 (T21) screening. Trials starting from 1984 identified several biomarkers with abnormal expression levels found in T21 pregnancies, including alpha-fetoprotein (AFP), pregnancy associated plasma protein A (PAPP-A), hCG and free β-hCG, etc [36–38]. It was only twenty years later when Wald et al. provided the first dataset based on a single cohort of women that empirically compared various combinations of screening markers in order to determine the option with the lowest false positive rate (FPR) [39]. Ultimately, this combination was identified as the Integrated Screening Test with a FPR of 0.9% at a 85% detection rate: a nuchal translucency and PAPP-A at 11 completed weeks of pregnancy and AFP, unconjugated uE3, hCG or free β-hCG, and inhibin-A in the early second trimester, which remains the most widely used screening protocol for T21.

With T21 as an example, composite biomarker panels demonstrate greater physiological relevance than a single biomarker; however, significant resources can be required to establish an optimized set of candidates. Additionally, while the development of the T21 biomarker panel did not utilize AI per se, it took over two decades of research before an optimized protocol was generally recognized. Machine learning’s ability for non-intuitive pattern recognition and to reconcile multimodal data has unlocked new opportunities for AI-driven predictors of cancer progression, and may significantly reduce the time needed to refine biomarker signatures of diagnostic value [40]. As such, the focus of this paper will be to investigate the use of such methods within the context of early detection of PDAC, with particular emphasis on its applications in liquid biopsy.

## Methods

A scoping review was conducted using established methodological frameworks and reported following the Preferred Reporting Items for Systematic Reviews and Meta-Analyses for Scoping Reviews (PRISMA-ScR) reporting guidelines [41, 42]. To identify potentially relevant documents, a search was performed using the PubMed and Scopus bibliographic databases with papers published from January 1, 2018 to July 11, 2025.

### Eligibility criteria

For this review, eligible papers needed to focus on the use of AI/ML in developing early cancer detection methodologies for PDAC using biomarkers found through liquid biopsies. Peer-reviewed journal papers were included if they were: (1) published between 2018 and 2025; (2) written in English; (3) involved human-derived biofluid samples (e.g. urine, serum, etc.) and described findings of PDAC-specific biomarkers. Papers were excluded if they: (1) did not implement AI/ML use into the study; (2) focused on neoplasms outside of pancreatic origin; (3) mainly described mechanistic determination of pathophysiology, or primarily explored therapies targeting PDAC.

### Information sources and search terms

To identify potentially relevant documents, the following bibliographic databases were searched from 2018 to 2025: PubMed and Scopus. The search strategies were drafted by an experienced librarian (P.B.), and further refined through team discussion. The final search results were exported into Zotero, and duplicates were removed. The studies were initially searched in PubMed using relevant keywords and Medical Subject Heading (MeSH) terms, before being adapted into appropriate syntax for the Scopus search. The final search strategy for both PubMed and Scopus can be found in Supplement 1.

### Data synthesis

Several data points were manually extracted from the studies included, such as study cohort size, biomarker sample source and type (found in Table 1), along with type of AI/ML model and reported performance metrics (Table 2 and Supplement 2 respectively). A thematic approach was used when performing data synthesis using the information from the included studies. Firstly, the types of biofluids selected for liquid biopsy and the features of their targeted biomarkers were discussed. Then, a description and summary of the AI/ML techniques used, along with the features extracted, model performances by each team are described. Microsoft Excel was used for storage and synthesized data management while Zotero was used for reference management.

**Table 1:** Characteristics of included studies with each study’s liquid biopsy source, cohort size and pathology type (PDAC, IPMN, CCA, etc.), and studied biomarkers (*n* = 18) [43–47, 49–53, 66–72]

Author	Sample type	Cohort size	Biomarker type
Cheng et al. [46]	Blood	PDAC (*n* = 32)	Circulating hybrid cells
Yang et al. [57]	Blood	PDAC (*n* = 89), non-disease control (n = 115)	Extracellular vesicular mRNA and miRNA, ccfDNA, CA19-9, mutant KRAS allele
Yuan et al. [43]	Serum	PDAC (*n* = 115), non-disease control (*n* = 2759)	Extracellular miRNA
Nakamura et al. [52]	Plasma, Serum	PDAC (*n* = 168), non-disease controls (*n* = 124)	Exosomal miRNA and cell-free miRNA
Zheng et al. [44]	Blood	PDAC (*n* = 57), breast cancer (*n* = 79), non-disease controls (*n* = 84)	Exosomes
Genco et al. [66]	Pancreatic cyst tissue and fluid, blood	Pancreatic precursor lesions (*n* = 47)	KRASmut DNA, MUC1, and CD55
Baba et al. [67]	Urine	PDAC (*n* = 153), non-disease controls (*n* = 309)	Extracellular vesicle miRNA
Hartwig et al. [68]	Plasma	PDAC (*n* = 40), IPMN (*n* = 7), chronic pancreatitis (*n* = 30), non-malignant controls (*n* = 26), healthy controls (*n* = 12)	ccfDNA
Shi et al. [53]	Serum, pancreatic and para-cancerous (healthy) tissue	PDAC (*n* = 24), IPMN (*n* = 1), NET (*n *= 1)	miRNA
Ito et al. [69]	Bile	CCA (*n* = 17), AC (*n* = 3), PDAC (*n* = 2), IPMN (*n* = 1), insulinoma (*n* = 1)	cfDNA
Zhu et al. [47]	Portal venous blood	PDAC (*n* = 39)	Circulating tumor cells
Zhao et al. [70]	Blood, PDAC tissue and para-cancerous tissue	PDAC (*n* = 262), healthy controls (*n *= 216)	ccfDNA
Xu et al. [49]	Plasma	PDAC (*n* = 20), pancreatitis (*n* = 20), healthy controls (*n* = 20)	Exosomal protein markers GPC1, MUC1, EGFR, and EphA2
Lee et al. [51]	Blood	PDAC (*n* = 417), non-disease controls (*n *= 1087)	15 mRNAs (CCL5, CCR5, CLEC7A, CXCL8, CXCR2, CXCR4, FOXP3, IFNA1, IFNL1, PTGES, PTGES2, PTGS2, SLC27A2, TNF, and VEGFA)
Shelly and Sivakumari [71]	Urine	PDAC (*n* = 199), benign (chronic pancreatitis/benign pancreatic cysts) (*n* = 208), healthy controls (*n* = 183)	Creatinine, LYVE1, REG1B, and TFF1
Greenberg et al. [72]	Plasma	PDAC (*n* = 6), healthy controls (*n* = 5)	Extracellular vesicle marker ATP6V0b
Eledkawy, Hamza, El-Metwally [45]	Plasma	PDAC (*n* = 93), normal controls (*n* = 812)	cfDNA/ctDNA
Angelioudaki et al. [50]	Blood	PDAC (*n* = 72), NET (*n* = 12), benign pancreatic lesions (*n* = 16), age-matched controls (*n* = 26)	Extracellular vesicles

*PDAC* pancreatic ductal adenocarcinoma, *IPMN* intraductal papillary mucinous neoplasm, *NET* pancreatic neuroendocrine tumor, *CCA* cholangiocarcinoma, *AC* ampullary carcinoma.

**Table 2:** Types of AI or ML techniques used for early detection of PDAC (*n* = 18).

Author	AI Approach	AI/ML Method(s)
Cheng et al. [46]	Hybrid AI (rule-based + ML)	Random Forest
Yang et al. [57]	ML	SVM (Support Vector Machine) or Support Vector Classifier, Logistic Regression, Linear Discriminant Analysis (LDA), k-Nearest Neighbors (k-NN), LASSO, Naïve Bayes
Yuan et al. [43]	ML	Random Forest, SVM (Support Vector Machine) or Support Vector Classifier, k-Nearest Neighbors (k-NN), Boruta, Decision Tree (including optimized, extra), mRMR (minimum Redundancy Maximum Relevance)
Nakamura et al. [52]	ML	Logistic Regression
Zheng et al. [44]	DL	Neural Networks
Genco et al. [66]	ML	k-Nearest Neighbors (k-NN), Principal Component Analysis (PCA)
Baba et al. [67]	ML	SVM (Support Vector Machine) or Support Vector Classifier
Hartwig et al. [68]	ML	SVM (Support Vector Machine) or Support Vector Classifier
Ito et al. [69]	ML	Random Forest
Shi et al. [53]	ML	Random Forest, SVM (Support Vector Machine) or Support Vector Classifier, Logistic Regression, CART (Classification and Regression Tree)
Zhao et al. [70]	ML	Random Forest, Boruta, Recursive Feature Elimination (RFE), Ensemble Bi-omics
Zhu et al. [47]	DL	Neural Networks
Angelioudaki et al. [50]	ML	Random Forest, SVM (Support Vector Machine) or Support Vector Classifier, Logistic Regression, XGBoost
Eledkawy et al. [45]	ML	Random Forest, XGBoost, Decision Tree (including optimized, extra), Quadratic Discriminant Analysis (QDA)
Greenberg et al. [72]	ML	Random Forest, Linear Discriminant Analysis (LDA), LASSO
Lee et al. [51]	ML	Random Forest, SVM (Support Vector Machine) or Support Vector Classifier, Logistic Regression, XGBoost, LightGBM
Shelly and Sivakumari [71]	ML	Decision Tree (including optimized, extra), Bagged Trees Ensemble
Xu et al. [49]	ML	Linear Discriminant Analysis (LDA)

*ML* machine learning, *DL* deep learning.

## Results

**Fig. 3:**
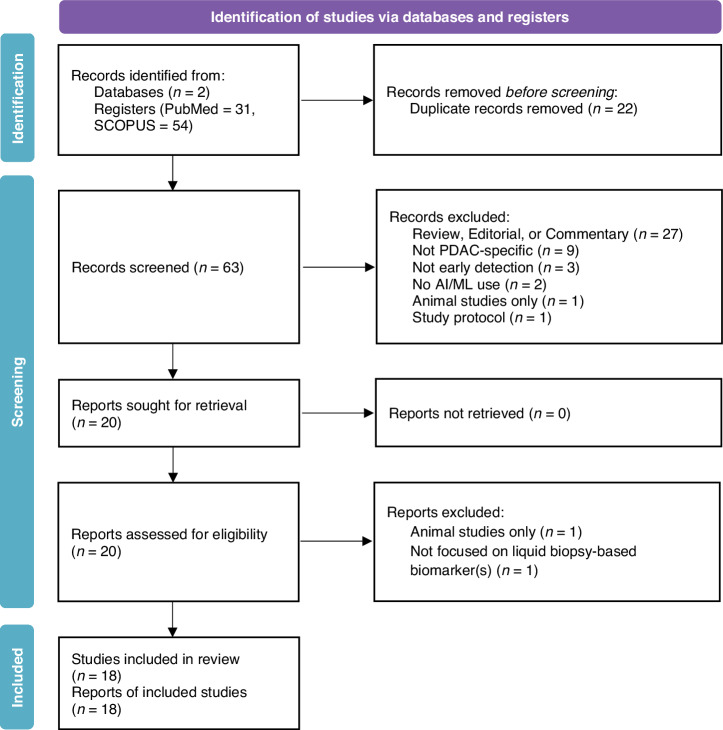
Preferred Reporting Items for Systematic Reviews and Meta-Analyses for Scoping Reviews (PRISMA-ScR) Flow Diagram outlining study selection process. Reasons for record exclusion ranged from being a review, editorial, or commentary article; not being PDAC-specific; not for early detection of PDAC; no stated use of AI/ML; exclusively focused on animal studies; and developing study protocol.

The initial search criteria outlined above yielded 85 studies from two publicly available online databases: PubMed (*n* = 31) and Scopus (*n* = 54). This was reduced to 63 studies after removing duplicates, down to 20 studies after title and abstract screening by two independent reviewers (J.K. and M.S.) based on eligibility criteria described above, and ultimately 18 following full-text review. A detailed breakdown is reported in the PRISMA flow diagram (Fig. 3). The list of papers included within the final review can also be found in Supplement 2.

### Key features of included studies

All 18 studies (Table 1) were published in peer-reviewed journals between the years 2020 and 2025, with two-thirds of the papers (*n* = 12) published in 2024 or 2025. The pathology types included disease states within the broader category of pancreatic and periampullary lesions, which are typically discussed together and were represented in the participant population studied. It should be noted that Yuan et al., Zheng et al., and Eledkawy et al. also included participants with non-pancreatic neoplasms such as breast, colorectal, and liver cancer [43–45]. The majority of liquid biopsy samples were blood (*n* = 15), while urine, bile, and pancreatic cyst fluid were identified for use in the detection of PDAC by one or two teams each.

### AI techniques employed

15 out of the 18 studies (Table 2) utilized supervised ML-based methodologies, while two studies used DL, e.g. fully connected neural networks. Additionally, Cheng et al. used a combination of what is described as “rule-based and machine learning techniques” [46]. The specific methods described by each paper are summarized in Table 2 (some papers utilized multiple methods within their final models), with the most common methods being random forest, support vector machines, and logistic regression.

For the studies using machine learning, output labels ranged from cancer versus healthy controls, differentiating between various types of cancer (PDAC, breast, colorectal), or PDAC versus benign pancreatic lesions. The features used include ccfDNA mutation profiles, miRNA expression, or EV surface markers. After training their models, teams move on to validation using unseen data samples to reduce overfitting. Many of the included studies used a cross-validation technique (5-fold or 10-fold), where the dataset is split into smaller folds before training and testing is repeated multiple times, while other studies reserved ~20–30% of the dataset for validation.

The two studies applying DL were trained on inputs such as CA19-9 levels, CTC counts, and predicted diagnostic categories such as CA19-9-negative PDAC or differentiating between breast and pancreatic cancer [44, 47]. Both studies performed validation using independent testing cohorts separate from the ones used for model training. Zheng et al. initially trained their dataset on 102 patients (66 PDAC, 36 healthy controls) before validating with another set of 70 patients (33 PDAC, 37 controls) [44]. Zhu et al. trained their DL model on a set of 39 PDAC patients before validation using a dataset of CA19-9-negative PDAC patients split 70/30 [47].

### Model performance

The papers present models with a wide range in their ability to detect PDAC across several clinical comparisons as seen in Supplement 2, reported as performance metrics. As patients with acute or chronic pancreatitis are at increased risk of acquiring PDAC, some groups have developed liquid biopsy biomarkers that acknowledge this observed course of disease progression [48]. Xu et al. established Exo-signature, a panel that provides high performance in distinguishing PDAC from both pancreatitis (AUC = 0.9188) and healthy controls (AUC = 0.9713) [49]. Angelioudaki et al. compare PDAC to benign lesions like pancreatic pseudocysts (96% accuracy with RF), which can often be misinterpreted on imaging [50].

Generally, architectures performed better with certain data modalities. In particular, miRNA-based models had both high accuracy (99.3% with SVM and 97.6% with kNN (Yuan et al., 2021); 94.4% with RF (Shi et al., 2024)) and AUC (0.92 with LR (Nakamura et al., 2022); 0.956 with SVM (Lee et al., 2025)) [51, 52, 53]. Meanwhile, other biomarker classes, e.g., exosomes and CTCs, underperformed across several model types. We hypothesize that this is due to inherent structural properties of miRNA. Often encapsulated, these short (18–22 nucleotides in length) sequences are protected from degradation by RNases and can be more easily isolated from biofluids using standardized molecular techniques [54]. On the other hand, EVs and exosomes remain challenging to isolate and purify, with no widely accepted standardized protocols at this time [55]. CTCs are also more fragile and rare in biological samples, while ctDNA is highly fragmented and susceptible to rapid clearance [54, 56]. As a result, more complex enrichment and purification approaches are required which can affect the overall reliability of the data generated from these biomarker types.

Combining CA19-9 with other biomarkers, as Yang et al. (2020) and Lee et al. (2025) do, improved model performance over using CA19-9 as a singular predictor of PDAC [51, 57]. Akin to the case of T21 screening, a composite panel may offer superior diagnostic accuracy by providing a more nuanced physiological representation of the patient.

## Discussion

Risk stratification is a critical component in the development of strategic screening protocols for PDAC, but still remains challenging. Multiple professional societies, ranging from the NCCN (National Comprehensive Cancer Network), CAPS (International Cancer of the Pancreas Screening Consortium), AGA (American Gastroenterological Association), ASGE (American Society for Gastrointestinal Endoscopy), and ACG (American College of Gastroenterology), have provided guidelines with varying concordance on specific eligibility criteria and implementation strategies [58, 59] for populations at high risk for PDAC [13].

As outlined in the 2020 AGA Clinical Practice Update on pancreatic cancer screening, these imaging-based methods put patients at risk of harm in several ways [14]. Specifically, EUS may result in tissue injury or adverse reactions to intravenous contrast or anesthesia. While MRCP is non-invasive, there are limitations in its specificity, particularly in differentiating between low- and high-grade dysplastic pancreatic precursor lesions. This increases the possibility of false positives, which may lead to unnecessary follow-up with more invasive procedures and their associated risks. Additionally, interval cancers, i.e. those which are diagnosed between standard screening timepoints, often present with rapid proliferation and worse prognosis than tumors detected during scheduled visits. Here, the suboptimal sensitivity rates can lead to concerns with false reassurances in vulnerable populations [12]. As such, the 2025 ASGE guidelines recommend that high-risk populations, such as those with genetic susceptibility, should receive PDAC screening on an annual basis [59].

Emerging evidence from prospective surveillance programs has demonstrated that systematic screening of high-risk individuals can detect earlier-stage disease with markedly improved survival outcomes. The Multicenter Cancer of Pancreas Screening Study (CAPS5) provides strong evidence for this shift, reporting that among 1461 high-risk individuals undergoing annual surveillance with EUS and/or MRI/MRCP, 77.8% of surveillance-detected PDACs were stage I by surgical pathology, compared to only 14.3% of cancers detected outside surveillance [60].

CAPS5 represents the greater ongoing efforts towards a paradigm shift in PDAC management with the goal of developing infrastructure for biomarker discovery, which serves to inform future screening methodologies. To this end, the Pancreatic Cancer Early Detection (PRECEDE) Consortium represents the largest international effort to date, enrolling over 3000 high-risk individuals across multiple continents with standardized data collection, imaging protocols, and biospecimen banking [61, 62].

Given these considerations and new developments, a gradual implementation of screening protocols based on precision medicine principles to prioritize individuals at high risk for PDAC may help mitigate unnecessary interventions and provide a stepwise progression in terms of procedural invasiveness. Using a personalized medicine framework, a consensus could be that the high-risk cohort would include patients with any established risk factors for PDAC, such as tobacco smoking, chronic pancreatitis, along with genetics and familial history. Moreover, the disproportionate rise in PDAC incidence among non-White women is concerning: these sociodemographic factors should also be acknowledged when developing equitable screening and surveillance protocols [4, 5].

### Limitations

17 out of the 18 studies in this review included cohorts of patients with confirmed cases of PDAC, which often reflects later-stage disease and therefore has potentially limited efficacy within the development of methods for early detection. At the same time, less than half of the studies included patients with precursor conditions such as chronic pancreatitis or benign pancreatic lesions, which could provide more relevant insights on the clinical landscape often seen prior to PDAC. Additionally, studies tended to be smaller in scale, with only three studies including more than 1000 participants. These three studies also focused on platforms identifying several types of malignancies, reducing the specific focus on PDAC and therefore shedding less light on the nuances (types of genetic mutations, tumor staging) of these cases at presentation.

With regards to the use of ML, the first limitation we observed was the lack of standardized reporting of model performance metrics. For the validation step, there was a wide variation in the types and number of values reported, including accuracy, sensitivity, specificity, and AUC as seen in Supplement 2. This variability makes comparing performance across different studies challenging, which could impact the ability to conduct systematic reviews in the future as the application of AI in liquid biopsy continues to increase. It is also important to note that neural networks with hidden layers essentially function as “black boxes”, making it difficult to monitor how predictions are being made, as unsupervised model training is a highly iterative and non-transparent process. In addition, a clear explanation of model architecture will be crucial for study validation and replication.

A limitation pertaining to this review is that our search strategy was primarily focused on studies explicitly framed within the “liquid biopsy” paradigm, as seen in Supplement 1. As a result, emerging microbiome-based approaches that do not use this terminology may have been excluded from discussion. In particular, recent studies have applied machine learning techniques to fecal and salivary microbiome profiles for early detection of PDAC, demonstrating promising diagnostic performance [63–65]. As microbiome-based diagnostics continue to evolve, future reviews incorporating broader search terms may further elucidate the role of AI/ML-driven microbial profiling as a complementary strategy for early pancreatic cancer detection.

The non- or minimally invasive liquid biopsy approach has the potential to ensure greater accessibility to PDAC surveillance, while also reducing the procedural risk to patients. We recognize that this is an emerging field with limited clinical implementation at the time of publication, and readily look forward to seeing how similar studies progress towards tangibly contributing to improving patient outcomes.

## Conclusion

Non- or minimally invasive screening via liquid biopsy for PDAC is a promising and viable complementary tool to existing imaging-based screening measures in order to mitigate the procedural burden on patients. Given the tendency of PDAC to remain asymptomatic for extended periods of time, the future of its early detection requires more proactive measures, such as regular surveillance. AI/ML unlocks the potential for multianalyte screening panels that could provide more robust diagnostic value compared to CA19-9 alone. With continued improvements, a PDAC panel may eventually be routinely available for patients, which can improve patient outcomes. By reducing the barrier to accessing timely screenings, it may be possible to reverse PDAC’s reputation as one of the most lethal common cancers in the US.

## Supplementary information

Supplement 1

Supplement 2

## Data Availability

Data sharing is not applicable to this article as no datasets were generated or analyzed during the current study.

## References

[1] Porta M, Fabregat X, Malats N, Guarner L, Carrato A, de Miguel A, et al. Exocrine pancreatic cancer: symptoms at presentation and their relation to tumour site and stage. Clin Transl Oncol. 2005;7:189–97. 10.1007/BF02712816. 15960930 10.1007/BF02712816

[2] Kalser MH, Barkin J, MacIntyre JM. Pancreatic cancer. Assessment of prognosis by clinical presentation. Cancer. 1985;56:397–402. 10.1016/S0140-6736(10)62307-0. 4005804 10.1002/1097-0142(19850715)56:2<397::aid-cncr2820560232>3.0.co;2-i

[3] Lasse Opsahl EL, Espinoza CE, Olivei AC, Okoye JO, Watkoske H, Hoffman MT, et al. Fibroblast STAT3 activation drives organ-specific premetastatic niche formation. Cancer Res. 2026;86:22–39. 10.1158/0008-5472.CAN-25-3472. 41105672 10.1158/0008-5472.CAN-25-3472 PMC12757723

[4] Moshayedi N, Escobedo AL, Thomassian S, Osipov A, Hendifar AE. Race, sex, age, and geographic disparities in pancreatic cancer incidence. J Clin Oncol. 2022;40:520–520. 10.1200/jco.2022.40.4_suppl.520.34878806

[5] Samaan JS, Abboud Y, Oh J, Jiang Y, Watson R, Park K, et al. Pancreatic cancer incidence trends by race, ethnicity, age and sex in the united states: a population-based study, 2000–2018. Cancers. 2023;15:870 10.3390/cancers15030870. 36765827 10.3390/cancers15030870 PMC9913805

[6] Bekkali NLH, Oppong KW. Pancreatic ductal adenocarcinoma epidemiology and risk assessment: could we prevent? Possibility for an early diagnosis. Endosc Ultrasound. 2017;6:S58–S61. 10.4103/eus.eus_60_17. 29387690 10.4103/eus.eus_60_17 PMC5774073

[7] Garajová I, Peroni M, Gelsomino F, Leonardi F. A simple overview of pancreatic cancer treatment for clinical oncologists. Curr Oncol. 2023;30:9587–601. 10.3390/curroncol30110694. 37999114 10.3390/curroncol30110694 PMC10669959

[8] Cancer of the Pancreas—Cancer Stat Facts. SEER. Accessed 11 July 2025. https://seer.cancer.gov/statfacts/html/pancreas.html.

[9] Agrawal R, Natarajan KN. Oncogenic signaling pathways in pancreatic ductal adenocarcinoma. Adv Cancer Res. 2023;159:251–83. 10.1016/bs.acr.2023.02.006. 37268398 10.1016/bs.acr.2023.02.006

[10] Li O, Li L, Sheng Y, Ke K, Wu J, Mou Y, et al. Biological characteristics of pancreatic ductal adenocarcinoma: Initiation to malignancy, intracellular to extracellular. Cancer Lett. 2023;574:216391 10.1016/j.canlet.2023.216391. 37714257 10.1016/j.canlet.2023.216391

[11] Stoffel EM, McKernin SE, Brand R, Canto M, Goggins M, Moravek C, et al. Evaluating susceptibility to pancreatic cancer: ASCO provisional clinical opinion. J Clin Oncol. 2019;37:153–64. 10.1200/JCO.18.01489. 30457921 10.1200/JCO.18.01489

[12] Blackford AL, Canto MI, Dbouk M, Hruban RH, Katona BW, Chak A, et al. Pancreatic cancer surveillance and survival of high-risk individuals. JAMA Oncol. 2024;10:1087–96. 10.1001/jamaoncol.2024.1930. 38959011 10.1001/jamaoncol.2024.1930 PMC11223057

[13] Jayakrishnan T, Ng K. Early-onset gastrointestinal cancers: a review. *JAMA*. 2025. 10.1001/jama.2025.10218. 10.1001/jama.2025.10218 PMC13281743 40674064

[14] Aslanian HR, Lee JH, Canto MI. AGA clinical practice update on pancreas cancer screening in high-risk individuals: expert review. Gastroenterology. 2020;159:358–62. 10.1053/j.gastro.2020.03.088. 32416142 10.1053/j.gastro.2020.03.088

[15] Ballehaninna UK, Chamberlain RS. Biomarkers for pancreatic cancer: promising new markers and options beyond CA 19-9. Tumor Biol. 2013;34:3279–92. 10.1007/s13277-013-1033-3. 10.1007/s13277-013-1033-3 23949878

[16] Zhao B, Zhao B, Chen F. Diagnostic value of serum carbohydrate antigen 19-9 in pancreatic cancer: a systematic review and meta-analysis. Eur J Gastroenterol Hepatol. 2022;34:891–904. 10.1097/MEG.0000000000002415. 35913776 10.1097/MEG.0000000000002415

[17] Borea R, Saldanha EF, Maheswaran S, Nicolo E, Singhal S, Pontolillo L, et al. Cancer in a drop: advances in liquid biopsy in 2024. Crit Rev Oncol Hematol. 2025;213:104776 10.1016/j.critrevonc.2025.104776. 40447209 10.1016/j.critrevonc.2025.104776

[18] Newman L, Rowland A. Detection and isolation of tissue-specific extracellular vesicles from the blood. J Extracell Biol. 2025;4:e70059 10.1002/jex2.70059. 40552103 10.1002/jex2.70059 PMC12183346

[19] Andre M, Caobi A, Miles JS, Vashist A, Ruiz MA, Raymond AD. Diagnostic potential of exosomal extracellular vesicles in oncology. BMC Cancer. 2024;24:322. 10.1186/s12885-024-11819-4. 38454346 10.1186/s12885-024-11819-4 PMC10921614

[20] Rayamajhi S, Sipes J, Tetlow AL, Saha S, Bansal A, Godwin AK. Extracellular vesicles as liquid biopsy biomarkers across the cancer journey: from early detection to recurrence. Clin Chem. 2024;70:206–19. 10.1093/clinchem/hvad176. 38175602 10.1093/clinchem/hvad176 PMC12374260

[21] Suárez B, Solé C, Márquez M, Nanetti F, Lawrie CH. Circulating microRNAs as cancer biomarkers in liquid biopsies. In: Schmitz U, Wolkenhauer O, Vera-González J, eds. *Systems biology of MicroRNAs in cancer*. Springer International Publishing; 2022:23–73. 10.1007/978-3-031-08356-3_2. 10.1007/978-3-031-08356-3_2 36352210

[22] Liu T, Chen Z, Chen W, Evans R, Xu J, Reeves ME, et al. Dysregulated miRNAs modulate tumor microenvironment associated signaling networks in pancreatic ductal adenocarcinoma. Precis Clin Med. 2023;6:pbad004 10.1093/pcmedi/pbad004. 37007745 10.1093/pcmedi/pbad004 PMC10052370

[23] Joosse SA, Pantel K. Circulating DNA and liquid biopsies in the management of patients with cancer. Cancer Res. 2022;82:2213–5. 10.1158/0008-5472.CAN-22-1405. 35702889 10.1158/0008-5472.CAN-22-1405

[24] Deng Z, Wu S, Wang Y, Shi D. Circulating tumor cell isolation for cancer diagnosis and prognosis. *eBioMedicine*. 2022;83. 10.1016/j.ebiom.2022.104237. 10.1016/j.ebiom.2022.104237 PMC9440384 36041264

[25] Pang TCY, Po JW, Becker TM, Goldstein D, Pirola RC, Wilson JS, et al. Circulating tumour cells in pancreatic cancer: A systematic review and meta-analysis of clinicopathological implications. Pancreatology. 2021;21:103–14. 10.1016/j.pan.2020.11.022. 33309014 10.1016/j.pan.2020.11.022

[26] Earl J, Garcia-Nieto S, Martinez-Avila JC, Montans J, Sanjuanbenito A, Rodríguez-Garrote M, et al. Circulating tumor cells (Ctc) and Kras mutant circulating free Dna (cfdna) detection in peripheral blood as biomarkers in patients diagnosed with exocrine pancreatic cancer. BMC Cancer. 2015;15:797. 10.1186/s12885-015-1779-7. 26498594 10.1186/s12885-015-1779-7 PMC4619983

[27] Lee JS, Han Y, Yun WG, Kwon W, Kim H, Jeong H, et al. Parallel analysis of pre- and postoperative circulating tumor DNA and matched tumor tissues in resectable pancreatic ductal adenocarcinoma: a prospective cohort study. Clin Chem. 2022;68:1509–18. 10.1093/clinchem/hvac153. 36177751 10.1093/clinchem/hvac153

[28] Keane F, Saadat LV, O’Connor CA, Chou JF, Bowman AS, Xu F, et al. Clinical utility and tissue concordance of circulating tumor DNA in pancreatic ductal adenocarcinoma. *J Natl Cancer Inst*. 2025:djaf139. 10.1093/jnci/djaf139. 10.1093/jnci/djaf139 PMC12415951 40511613

[29] Tsui WHA, Ding SC, Jiang P, Lo YMD. Artificial intelligence and machine learning in cell-free-DNA-based diagnostics. Genome Res. 2025;35:1–19. 10.1101/gr.278413.123. 39843210 10.1101/gr.278413.123 PMC11789496

[30] Breiman L. Random forests. Mach Learn. 2001;45:5–32. 10.1023/A:1010933404324.

[31] Noble WS. What is a support vector machine. Nat Biotechnol. 2006;24:1565–7. 10.1038/nbt1206-1565. 17160063 10.1038/nbt1206-1565

[32] Tran KA, Kondrashova O, Bradley A, Williams ED, Pearson JV, Waddell N. Deep learning in cancer diagnosis, prognosis and treatment selection. Genome Med. 2021;13:152. 10.1186/s13073-021-00968-x. 34579788 10.1186/s13073-021-00968-x PMC8477474

[33] Li J, Shi J, Chen J, Du Z, Huang L. Self-attention random forest for breast cancer image classification. Front Oncol. 2023;13. 10.3389/fonc.2023.1043463. 10.3389/fonc.2023.1043463 PMC9939756 36814814

[34] Velazquez M, Lee Y. Random forest model for feature-based Alzheimer’s disease conversion prediction from early mild cognitive impairment subjects. PLoS ONE. 2021;16:e0244773 10.1371/journal.pone.0244773. 33914757 10.1371/journal.pone.0244773 PMC8084194

[35] Goh KH, Wang L, Yeow AYK, Poh H, Li K, Yeow JJL, et al. Artificial intelligence in sepsis early prediction and diagnosis using unstructured data in healthcare. Nat Commun. 2021;12:711. 10.1038/s41467-021-20910-4. 33514699 10.1038/s41467-021-20910-4 PMC7846756

[36] Brock DJ, Barron L, Holloway S, Liston WA, Hillier SG, Seppala M. First-trimester maternal serum biochemical indicators in Down syndrome. Prenat Diagn. 1990;10:245–51. 10.1002/pd.1970100406. 1694994 10.1002/pd.1970100406

[37] Brizot ML, Jauniaux E, Mckie AT, Farzaneh F, Nicolaides KH. Molecular interactions during pregnancy: Placental expression of α and β subunits of human chorionic gonadotrophin in early pregnancies with Down’s syndrome. Hum Reprod. 1995;10:2506–9. 10.1093/oxfordjournals.humrep.a136331. 8530698 10.1093/oxfordjournals.humrep.a136331

[38] Newby D, Aitken DA, Crossley JA, Howatson AG, Connor JM. The pathophysiology of Down’s syndrome pregnancies. Early Hum Dev. 1996;47:S67–S68. 10.1016/S0378-3782(96)01824-5. 9031845 10.1016/s0378-3782(96)01824-5

[39] Wald NJ, Rodeck C, Hackshaw AK, Rudnicka A. SURUSS in perspective. BJOG Int J Obstet Gynaecol. 2004;111:521–31. 10.1111/j.1471-0528.2004.00193.x. 10.1111/j.1471-0528.2004.00193.x 15198778

[40] Alum EU. AI-driven biomarker discovery: enhancing precision in cancer diagnosis and prognosis. Discov Oncol. 2025;16:313 10.1007/s12672-025-02064-7. 40082367 10.1007/s12672-025-02064-7 PMC11906928

[41] Levac D, Colquhoun H, O’Brien KK. Scoping studies: advancing the methodology. Implement Sci. 2010;5:69 10.1186/1748-5908-5-69. 20854677 10.1186/1748-5908-5-69 PMC2954944

[42] Tricco AC, Lillie E, Zarin W, O’Brien KK, Colquhoun H, Levac D, et al. PRISMA extension for scoping reviews (PRISMA-ScR): checklist and explanation. Ann Intern Med. 2018;169:467–73. 10.7326/M18-0850. 30178033 10.7326/M18-0850

[43] Yuan F, Li Z, Chen L, Zeng T, Zhang YH, Ding S, et al. Identifying the signatures and rules of circulating extracellular MicroRNA for distinguishing cancer subtypes. Front Genet. 2021;12:651610 10.3389/fgene.2021.651610. 33767734 10.3389/fgene.2021.651610 PMC7985347

[44] Zheng H, Zhao J, Wang X, Yan S, Chu H, Gao M, et al. Integrated pipeline of rapid isolation and analysis of human plasma exosomes for cancer discrimination based on deep learning of MALDI-TOF MS fingerprints. Anal Chem. 2022;94:1831–9. 10.1021/acs.analchem.1c04762. 35025210 10.1021/acs.analchem.1c04762

[45] Eledkawy A, Hamza T, El-Metwally S. Towards precision oncology: a multi-level cancer classification system integrating liquid biopsy and machine learning. *BioData Min*. 2025;18. 10.1186/s13040-025-00439-8 10.1186/s13040-025-00439-8 PMC11987386 40217526

[46] Cheng KS, Pan R, Pan H, Li B, Meena SS, Xing H, et al. ALICE: a hybrid AI paradigm with enhanced connectivity and cybersecurity for a serendipitous encounter with circulating hybrid cells. Theranostics. 2020;10:11026–48. 10.7150/thno.44053. 33042268 10.7150/thno.44053 PMC7532685

[47] Zhu Z, Zhang Y, Zhang W, Tang D, Zhang S, Wang L, et al. High-throughput enrichment of portal venous circulating tumor cells for highly sensitive diagnosis of CA19-9-negative pancreatic cancer patients using inertial microfluidics. Biosens Bioelectron. 2024;259:116411 10.1016/j.bios.2024.116411. 38781696 10.1016/j.bios.2024.116411

[48] Gandhi S, de la Fuente J, Murad MH, Majumder S. Chronic pancreatitis is a risk factor for pancreatic cancer, and incidence increases with duration of disease: a systematic review and meta-analysis. Clin Transl Gastroenterol. 2022;13:e00463 10.14309/ctg.0000000000000463. 35142721 10.14309/ctg.0000000000000463 PMC8963838

[49] Xu LL, Wang M, Wang YK, Chen YJ, Zhang YX, Zhang YQ, et al. Vessel-Like Microtunnels with Biomimetic Octopus Tentacles for Seizing and Detecting Exosomes to Diagnose Pancreatic Cancer. Small Weinh Bergstr Ger. 2025;21:e2502763. 10.1002/smll.202502763. 10.1002/smll.202502763 40424013

[50] Angelioudaki I, Iosif A, Kourou K, Tzingounis AG, Kigka V, Skreka AM, et al. A machine-learning approach for pancreatic neoplasia classification based on plasma extracellular vesicles. Front Oncol.2025;15. 10.3389/fonc.2025.1540195. 10.3389/fonc.2025.1540195 PMC12061713 40352592

[51] Lee JC, Kang SW, Sim EJ, Bae JS, Koo SM, Byoun MS, et al. Novel mRNA biomarker-based liquid biopsy for the detection of resectable pancreatic cancer. BMC Cancer. 2025;25:762 10.1186/s12885-025-14124-w. 40269781 10.1186/s12885-025-14124-w PMC12016232

[52] Nakamura K, Zhu Z, Roy S, Jun E, Han H, Munoz RM, et al. An Exosome-based Transcriptomic Signature for Noninvasive, Early Detection of Patients With Pancreatic Ductal Adenocarcinoma: AMulticenter Cohort Study. Gastroenterology. 2022;163:1252–1266.e2. 10.1053/j.gastro.2022.06.090. 35850192 10.1053/j.gastro.2022.06.090 PMC9613527

[53] Shi W, Wartmann T, Accuffi S, Al-Madhi S, Perrakis A, Kahlert C, et al. Integrating a microRNA signature as a liquid biopsy-based tool for the early diagnosis and prediction of potential therapeutic targets in pancreatic cancer. Br J Cancer. 2024;130:125–134. 10.1038/s41416-023-02488-4. 37950093 10.1038/s41416-023-02488-4 PMC10781694

[54] Gahlawat AW, Witte T, Sinn P, Schott S. Circulating cf-miRNA as a more appropriate surrogate liquid biopsy marker than cfDNA for ovarian cancer. Sci Rep. 2023;13:5503. 10.1038/s41598-023-32243-x. 37015943 10.1038/s41598-023-32243-x PMC10073086

[55] Sidhom K, Obi PO, Saleem A. A review of exosomal isolation methods: is size exclusion chromatography the best option. Int J Mol Sci. 2020;21:6466 10.3390/ijms21186466. 32899828 10.3390/ijms21186466 PMC7556044

[56] Leong SM, Tan KML, Chua HW, Huang MC, Cheong WC, Li MH, et al. Paper-based microRNA expression profiling from plasma and circulating tumor cells. Clin Chem. 2017;63:731–41. 10.1373/clinchem.2016.264432. 28073899 10.1373/clinchem.2016.264432

[57] Yang Z, LaRiviere MJ, Ko J, Till JE, Christensen T, Yee SS, et al. A Multianalyte Panel Consisting of Extracellular Vesicle miRNAs and mRNAs, cfDNA, and CA19-9 Shows Utility for Diagnosis and Staging of Pancreatic Ductal Adenocarcinoma. Clin Cancer Res Off J Am Assoc Cancer Res. 2020;26:3248–3258. 10.1158/1078-0432.CCR-19-3313. 10.1158/1078-0432.CCR-19-3313 PMC7334066 32299821

[58] NCCN Clinical Practice Guidelines in Oncology—Genetic/­familial high-risk assessment: breast, ovarian, pancreatic, and prostate. Published online February 19, 2026. Accessed 24 February 2026. https://www.nccn.org/professionals/physician_gls/pdf/genetics_bopp.pdf

[59] Sawhney MS, Calderwood AH, Thosani NC, Rebbeck TR, Wani S, Canto MI, et al. ASGE guideline on screening for pancreatic cancer in individuals with genetic susceptibility: summary and recommendations. Gastrointest Endosc. 2022;95:817–26. 10.1016/j.gie.2021.12.001. 35183358 10.1016/j.gie.2021.12.001

[60] Dbouk M, Katona BW, Brand RE, Chak A, Syngal S, Farrell JJ, et al. The multicenter cancer of pancreas screening study: impact on stage and survival. J Clin Oncol. 2022;40:3257–66. 10.1200/JCO.22.00298. 35704792 10.1200/JCO.22.00298 PMC9553376

[61] Haimi I, Zogopoulos G, Dettwyler SA, Everett JN, Bi Y, Brand RE, et al. Pancreatic imaging findings from the PRECEDE study: a large high-risk heritable cohort for pancreatic cancer. J Clin Oncol. 2023;41:689 10.1200/JCO.2023.41.4_suppl.689.

[62] Huang C, Simeone DM, Luk L, Hecht EM, Khatri G, Kambadakone A, et al. Standardization of MRI screening and reporting in individuals with elevated risk of pancreatic ductal adenocarcinoma: consensus statement of the PRECEDE Consortium. Am J Roentgenol. 2022;219:903–14. 10.2214/AJR.22.27859. 35856454 10.2214/AJR.22.27859

[63] Kartal E, Schmidt TSB, Molina-Montes E, Rodríguez-Perales S, Wirbel J, Maistrenko OM, et al. A faecal microbiota signature with high specificity for pancreatic cancer. Gut. 2022;71:1359–72. 10.1136/gutjnl-2021-324755. 35260444 10.1136/gutjnl-2021-324755 PMC9185815

[64] Lee D, Lee C, Han K, Goo T, Kim B, Han Y, et al. Machine learning models for pancreatic cancer diagnosis based on microbiome markers from serum extracellular vesicles. Sci Rep. 2025;15:10995. 10.1038/s41598-025-94183-y. 40164714 10.1038/s41598-025-94183-y PMC11958759

[65] Chen Y, Nian F, Chen J, Jiang Q, Yuan T, Feng H, et al. Metagenomic microbial signatures for noninvasive detection of pancreatic cancer. Biomedicines. 2025;13:1000 10.3390/biomedicines13041000. 40299688 10.3390/biomedicines13041000 PMC12025148

[66] Genco E, Modena F, Sarcina L, Björkström K, Brunetti C, Caironi M, et al. A Single-Molecule Bioelectronic Portable Array for Early Diagnosis of Pancreatic Cancer Precursors. Adv Mater DeerfieldBeach Fla. 2023;35:e2304102 10.1002/adma.202304102. 10.1002/adma.202304102 37452695

[67] Baba S, Kawasaki T, Hirano S, Nakamura T, Asano T, Okazaki R, et al. A noninvasive urinary microRNA-based assay for the detection of pancreatic cancer from early to late stages: a case control study. EClinicalMedicine. 2024;78:102936 10.1016/j.eclinm.2024.102936. 39764541 10.1016/j.eclinm.2024.102936 PMC11701472

[68] Hartwig C, Müller J, Klett H, Kouhestani D, Mittelstädt A, Anthuber A, et al. Discrimination of pancreato-biliary cancer and pancreatitis patients by non-invasive liquid biopsy. Mol Cancer. 2024;23:28 10.1186/s12943-024-01943-x. 38308296 10.1186/s12943-024-01943-x PMC10836044

[69] Ito S, Ando M, Aoki S, Soma S, Zhang J, Hirano N, et al. Usefulness of multigene liquid biopsy of bile for identifying driver genes of biliary duct cancers. Cancer Sci. 2024;115:4054–4063. 10.1111/cas.16365. 39377143 10.1111/cas.16365 PMC11611759

[70] Zhao G, Jiang R, Shi Y, Gao S, Wang D, Li Z, et al. Circulating cell-free DNA methylation-based multi-omics analysis allows early diagnosis of pancreatic ductal adenocarcinoma. Mol Oncol. 2024;18:2801–2813. 10.1002/1878-0261.13643. 38561976 10.1002/1878-0261.13643 PMC11547243

[71] Shelly M, Sivakumari S. Enhancing pancreatic cancer diagnostics: Ensemble-based model for automated urine biomarker classification. Comput Biol Med. 2025;189:109997. 10.1016/j.compbiomed.2025.109997. 40068492 10.1016/j.compbiomed.2025.109997

[72] Greenberg ZF, Ali S, Brock A, Jiang J, Schmittgen TD, Han S, et al. Nanomaterial isolated extracellular vesicles enable high precision identification of tumor biomarkers for pancreatic cancer liquid biopsy. JNanobiotechnology. 2025;23. 10.1186/s12951-025-03527-3. 10.1186/s12951-025-03527-3 PMC12211367 40598203

