# Supplementary material for: Machine learning and artificial intelligence in liquid biopsy-based early detection of pancreatic cancer: a scoping review
Source: BJC Rep. 2026 May 21;4:26. doi: 10.1038/s44276-026-00232-y (PMC13195041; doi:10.1038/s44276-026-00232-y)
Supplement: Supplementary file 1 — Supplement 1 [file 44276_2026_232_MOESM1_ESM.docx]

**Final Search Strategy**

Literature Search performed: July 11, 2025

**PubMed Search Terms**

((((Liquid biopsy[mesh] OR liquid biopsy[tiab] OR nanoliquid biopsy[tiab])) AND (((Circulating tumor DNA[mesh] OR neoplastic cells, circulating[mesh] OR microRNAs[mesh] OR Tumor DNA[tiab] OR neoplastic cells[tiab] OR microRNA[tiab] OR miRNA[tiab]) OR ((Biomarkers, tumors[mesh] OR tumor biomarkers[tiab]))) OR (Extracellular vesicles[mesh] OR extracellular vesicles[tiab] OR exosomes[tiab]))) AND (artificial intelligence[mesh:noexp] OR machine learning[mesh] OR artificial intelligence[tiab] OR machine learning[tiab] OR deep learning[tiab])) AND (pancreatic neoplasms[mesh] OR Pancreatic cancer[tiab] OR pancreatic neoplasms[tiab] OR pancreatic tumor[tiab] OR (pancreas[tiab] AND cancer[tiab]) OR (pancreas AND neoplasm[tiab]) OR (pancreas[tiab] AND tumor[tiab]))s

**Scopus Search Terms**

TITLE-ABS-KEY(“Liquid biopsy” OR “nanoliquid biopsy”) AND TITLE-ABS-KEY(“Circulating tumor DNA” OR “neoplastic cells circulating” OR “microRNAs” OR “Tumor DNA” OR “neoplastic cells” OR “microRNA” OR “miRNA” OR “tumor biomarkers” OR “Extracellular vesicles” OR “extracellular vesicles” OR “exosomes”) AND TITLE-ABS-KEY(“artificial intelligence” OR “machine learning” OR “deep learning”) AND TITLE-ABS-KEY(“pancreatic neoplasms” OR “Pancreatic cancer” OR “pancreatic neoplasms” OR “pancreatic tumor” OR “pancreas” AND “cancer” OR “pancreas” AND “neoplasm” OR “pancreas” AND “tumor”)
